# Improved Precision and Sensitivity: Refinement of a Homebrew Simoa Assay for Detecting pTau181 in Plasma and Serum

**DOI:** 10.1002/alz.095453

**Published:** 2025-01-09

**Authors:** Jeroen Vanbrabant, Sherif Bayoumy, Daniel Antwi‐Berko, Wiesje M. van der Flier, Inge M.W. Verberk, Charlotte Teunissen, Erik Stoops

**Affiliations:** ^1^ ADx NeuroSciences NV, Ghent Belgium; ^2^ Neurochemistry Laboratory, Department of Laboratory Medicine, Amsterdam Neuroscience, Vrije Universiteit Amsterdam, Amsterdam UMC, Amsterdam Netherlands; ^3^ Neurochemistry Laboratory, Department of Laboratory Medicine, Amsterdam UMC, Vrije Universiteit Amsterdam, Amsterdam Neuroscience, Amsterdam Netherlands; ^4^ Alzheimer Center, Department of Neurology, Amsterdam UMC, Vrije Universiteit Amsterdam, Amsterdam Neuroscience, Amsterdam Netherlands

## Abstract

**Background:**

Plasma tau phosphorylated at Threonine181 (pTau181) has been extensively studied as an accessible biomarker of amyloid pathology and is compared in various studies with other pTau epitopes. ADx has developed a Quanterix Homebrew Simoa assay using a highly pTau181 specific capture mAb (ADx252) and an N‐terminal tau detector ADx204 demonstrating high clinical performance (Janelidze *et al*., 2023; Ashton *et al*., 2022; Bayoumy *et al*., 2021). Nevertheless, measuring pTau181 in samples from young adults or serum that have lower levels remains challenging due to limited sensitivity and precision in the low calibrator range. This work describes an updated Homebrew assay for detection of pTau181 in human plasma and serum with increased precision and sensitivity compared to the previously published ADx pTau181 Simoa assay.

**Method:**

The ADx pTau181 assay was updated using re‐engineered formats of ADx252 (RD‐070) and ADx204 (RD‐073). Antibody conjugation to beads was optimized by C‐terminal incorporation of a tag to enhance antibody conjugation efficiency and robustness. The assay was further optimized using alternative diluents resulting in a linear curve over the full calibrator range. The clinical performance of the assay format was compared to the previous pTau181 assay version using plasma from 50 AD patients (defined by CSF biomarker profile) and 50 cognitively healthy donors from the Dutch Brain Research Registry (Hersenonderzoek.nl). Paired plasma and serum from a prospective collection of healthy donors (n = 50) were also analyzed using the optimized pTau181 assay.

**Result:**

The optimized assay showed a higher AUC of 0.96 (95% CI 0.92‐0.99) compared to 0.88 (95% CI 0.82 – 0.95) for the previous version (ΔAUC = 0.08, DeLong’s p = 0.047). Furthermore, our optimized assay was able to measure serum pTau 181, exhibiting a strong correlation of the analyte between plasma and serum and with pTau217 levels. Additionally, precision and sensitivity were improved (no vs. 18 plasma samples with CV%>20 on duplicate measurements; mean plasma sample S/N increased from 4.3 to 9.8).

**Conclusion:**

Our study illustrates the successful development of an optimized Homebrew pTau181 assay, with enhanced analytical and clinical performance achieved by an improved sensitivity and precision at measuring pTau 181 in plasma and serum samples.

